# Establishment of a Cynomolgus Macaque Model of Human T-Cell Leukemia Virus Type 1 (HTLV-1) Infection by Direct Inoculation of Adult T-Cell Leukemia Patient-Derived Cell Lines for HTLV-1 Infection

**DOI:** 10.1128/jvi.01339-22

**Published:** 2022-10-31

**Authors:** Emiko Urano, Kayoko Ueda, Mahoko Higuchi, Mugi Furukawa, Tomotaka Okamura, Yuetsu Tanaka, Yasuhiro Yasutomi

**Affiliations:** a Laboratory of Immunoregulation and Vaccine Research, Tsukuba Primate Research Center, National Institutes of Biomedical Innovation, Health and Nutrition, Tsukuba, Ibaraki, Japan; b Laboratory of Hematoimmunology, Graduate School of Health Sciences, Faculty of Medicine, University of the Ryukyus, Nishihara, Okinawa, Japan; c Division of Immunoregulation, Department of Molecular and Experimental Medicine, Mie University Graduate School of Medicine, Tsu, Mie, Japan; Ulm University Medical Center

**Keywords:** ATL, HTLV-1, cynomolgus macaque, nonhuman primate

## Abstract

Human T-cell leukemia virus type 1 (HTLV-1) is the causative agent of adult T-cell leukemia (ATL) and HTLV-1-associated myelopathy/tropical spastic paraparesis (HAM/TSP). However, the precise mechanisms leading to HTLV-1 chronic infection and the onset of the diseases have remained unclear, and effective vaccines for inhibiting the infection and the progression of pathogenesis have therefore not been developed. The use of a nonhuman primate (NHP) model is thought to be important for revealing the mechanisms of the progressive status and for the development of prevention procedures. In this study, we developed a cynomolgus macaque (CM) model of HTLV-1 infection by direct intravenous inoculation of HTLV-1-producing cells derived from ATL patients. The cell line used for infection, ATL-040, was selected as the most infectious one in our cell line library. CMs inoculated intravenously with 1 × 10^8^ ATL-040 cells per animal became persistently infected with HTLV-1, as shown by the HTLV-1 provirus load (PVL) in peripheral blood mononuclear cells and HTLV-1-specific antibodies (2/2 animals). One CM inoculated intravenously with 1 × 10^7^ ATL-040 cells did not have detectable PVLs despite the fact that anti-HTLV-1 antibodies were maintained for more than 2 years. Furthermore, immunological approaches, including CD8^+^ T cell depletion prior to infection (3/3 animals) and intrathecal inoculation (3/3 animals), led to increased proviral loads in the cynomolgus monkeys. The present method and the cynomolgus monkey model of HTLV-1 infection will be beneficial for immunological and virological studies on HTLV-1 aiming at the development of anti-HTLV-1 prophylactic vaccines and therapy drugs.

**IMPORTANCE** HTLV-1 was discovered in the 1980s as the causative agent of adult T-cell leukemia and HTLV-1-associated myelopathy/tropical spastic paraparesis. However, the precise mechanisms leading to HTLV-1 chronic infection and the onset of the diseases still remain unidentified. Thus, no effective vaccines to inhibit the infection and the progressive of pathogenesis have been developed. The use of appropriate animal models is essential for understanding HTLV-1 infection and pathogenesis. In order to establish a new nonhuman primate model for studies on HTLV-1 infection, cynomolgus monkeys were infected with HTLV-1 under a variety of experimental conditions. Our method, using a cell line generated from an ATL patient as a source of HTLV-1, was able to establish HTLV-1 infection in monkeys with a 100% success rate. This cynomolgus macaque model of HTLV-1 infection will contribute to the elucidation of HTLV-1 infection and its associated disease development.

## INTRODUCTION

Human T-cell leukemia virus type 1 (HTLV-1), the first human retrovirus to be discovered, is the infectious pathogen that causes adult T-cell leukemia (ATL) (1–3) and HTLV-1-associated myelopathy/tropical spastic paraparesis (HAM/TSP) (4, 5). Effective vaccines having the ability to escape from these diseases have not been developed. Only about 5% of HTLV-1-infected individuals (carriers) develop ATL or HAM/TSP (6–9). The development of ATL requires long-term chronic infection, since most ATL patients are infected with HTLV-1 in infancy by vertical transmission, mainly through breast feeding (10–12). In contrast, the development of HAM/TSP probably does not need such a long latent-infection period (10, 13). However, the precise mechanisms leading to HTLV-1 chronic infection and the onset of the diseases still remain unclear.

The host immune system is considered to control HTLV-1 infection like other chronic viral infections (14–16). It has been suggested that an immunocompromised condition is associated with the development of ATL and HAM/TSP (17, 18). Allogeneic hematopoietic stem cell transplantation has been used to improve the prognosis of aggressive ATL (19–22). However, the administration of immunosuppressive drugs during organ transplantation led to the subsequent development of ATL in some carriers who had other diseases with surgical treatment options (23–25). Furthermore, it was reported that HTLV-1-uninfected recipients became infected with HTLV-1 after kidney transplantation from carrier donors and then developed HAM/TSP progressively with a high incidence under the use of immunosuppressive drugs (26). Thus, analysis of the immune regulation of HTLV-1 infection *in vivo* is important for understanding the disease development. An animal model that has an intact immune system might be useful for revealing the host immune responses against HTLV-1 infection and how this virus establishes a lifelong persistent infection *in vivo*.

Animal models of human infectious diseases are important for elucidation of the pathogenesis and for the development of vaccines and therapeutic interventions. A nonhuman primate (NHP) model is an excellent model for studying human infectious diseases because NHPs share almost all of the relevant characteristics with humans (27). An NHP model of HTLV-1 infection is also considered to be an ideal model for revealing the mechanisms of disease progression and for developing preventive applications. So far, the major obstacle for the development of NHP models of HTLV-1 infection has been a lack of an efficient method of HTLV-1 infection. The first NHP model of HTLV-1 infection was reported using Japanese monkeys inoculated with a rabbit cell line producing HTLV-1 (28), followed by several trials using autologous HTLV-1-tranformed cells (29, 30). For example, (i) Nakamura et al. and Kazanji et al. reported that cynomolgus macaques (CMs) and squirrel monkeys can be infected with HTLV-1 by inoculating autologous HTLV-1-infected cells transformed by a human T cell line, MT-2, that continuously produces a large amount of HTLV-1 (29, 31). (ii) Beilke et al. reported that inoculation of autologous HTLV-1_KT_-infected cells derived from HAM/TSP patient resulted in persistent HTLV-1 infection in rhesus macaques (30). Finally, (iii) Kazanji et al. reported sustained HTLV-1 production in squirrel monkeys by using autologous and homologous HTLV-1-infected cells but not by using heterologous MT-2 cells (31). The HTLV-1-producing MT-2 cells have been commonly used in HTLV-1 research and in NHP models (32). However, an HTLV-1-infected NHP model cannot be easily established by traditional methods because (i) the success rate of transformation by MT-2 cells is not sufficient, (ii) establishment of autologous cells takes 2 to 4 months and then another 2 to 4 months are required for propagation to obtain a sufficient amount of cells for infection in an NHP if autologous transformed cells have been established, and (iii) infection cannot be established by direct intravenous injection of MT-2 cells in an NHP (31, 33). An HTLV-1-infected NHP model can be established if these difficulties are overcome, since NHPs are susceptible to HTLV-1.

In the present study, we established an NHP model of HTLV-1 infection using CMs as the host and a novel cell line generated from an ATL patient as the source of HTLV-1. CMs were selected because of their human-like characteristics, including higher brain functions, long life span, and single pregnancies, that are not found in other experimental animals. In addition, the body size of CMs is smaller than that of rhesus and Japanese macaques, and CMs are therefore easier to handle and more useful for pharmaceutical evaluation. In the present study, we established a CM model of persistent HTLV-1 infection by a novel method using ATL patient-derived cell lines.

## RESULTS

### Selection of a highly infectious HTLV-1-producing cell line.

**FIG 1:**
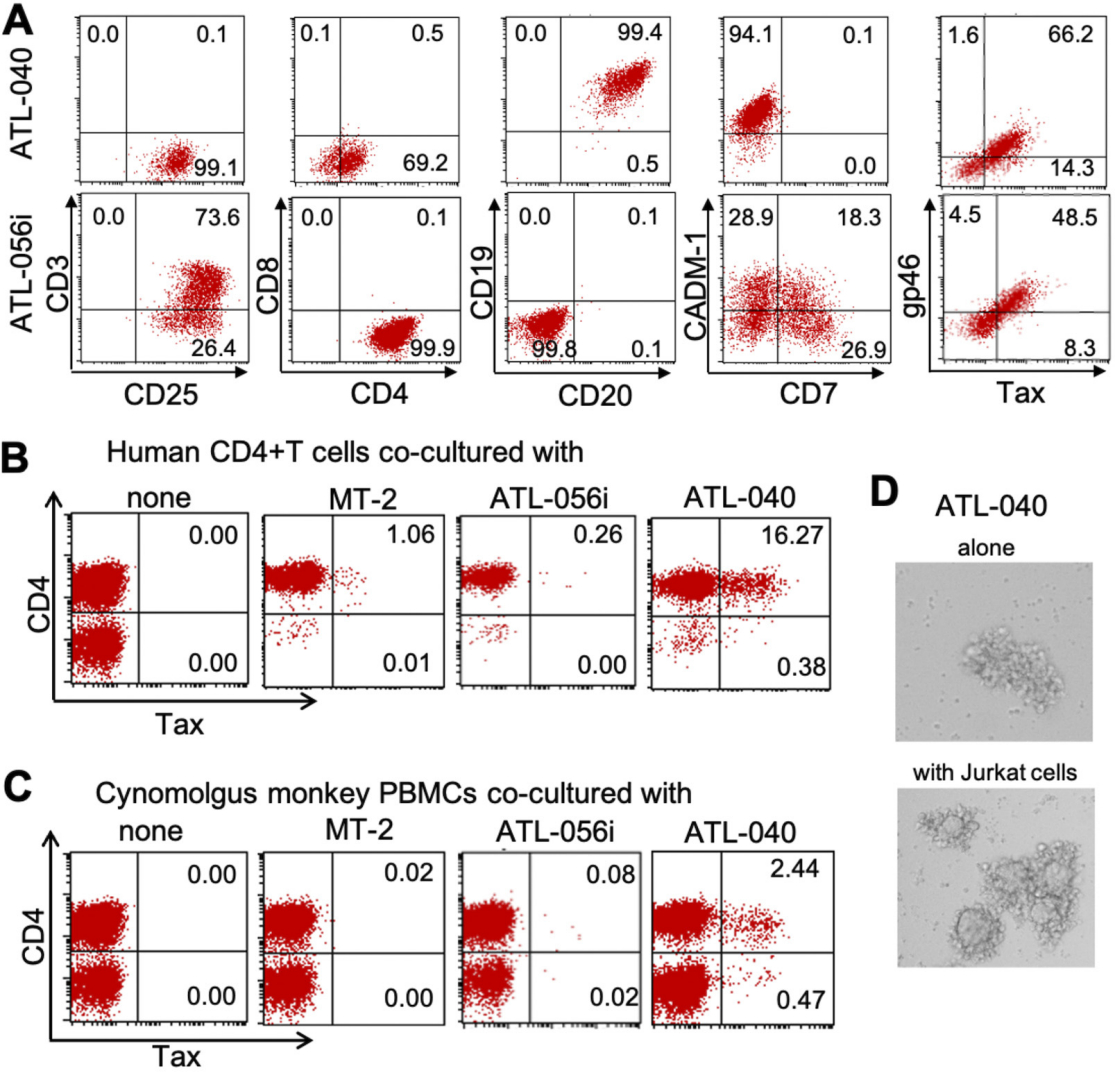
Phenotypes of HTLV-1-producing cells. (A) Characterization of ATL-040 (top) and ATL-056i (bottom) cells. Cells were stained for the indicated cell surface makers and intracellular Tax and Env gp46 antigens. (B, C) Human PBMCs (B) and CM PBMCs (C) were cocultured with various MMC-treated HTLV-1-producing cells for 2 weeks. Then, Tax antigen-expressing cells were quantitated by FCM. The numbers show the percentages of gated cells. (D) Syncytium formation ability of ATL-040 cells. ATL-040 cells were cultured alone or cocultured with Jurkat cells in 96-well U-bottom culture plates for 4 h, and syncytium formation was determined microscopically at a magnification of ×100.

In order to select the most appropriate cell line for HTLV-1 infection in monkeys, we screened our HTLV-1-producing cell lines that had been established in our laboratory from ATL patients and asymptomatic carriers (34) for their HTLV-1 transmission abilities using an HTLV-1-transformation assay (35), and selected two representative cell lines, ATL-056i and ATL-040. Both cell lines were established from an ATL patient’s peripheral blood mononuclear cells (PBMCs), cultured with or without interleukin-2 (IL-2) supplementation. The phenotypes of these cell lines were analyzed by flow cytometric analysis (FCM), including HTLV-1 envelope glycoprotein gp46 and transcription factor Tax expression (Fig. 1). The selected cell line, ATL-040, is an IL-2-independent CD19^+^ CD20^+^ B cell line, and ATL-056i is an IL-2-dependent CD4^+^ T cell line (Fig. 1A). First, we examined the transformation ability of these cell lines using human and cynomolgus macaque (CM) PBMCs by intracellular Tax FCM. As shown by the results in Fig. 1B and C, the ATL-040 cell line was superior to ATL-056i and MT-2 cells in efficient HTLV-1 transmission with both human PBMCs and CM peripheral PBMCs. In accordance with this result, ATL-040 cells were capable of generating large syncytia when cocultured with HTLV-1-negative Jurkat cells (Fig. 1D), in contrast to both MT-2 cells, which did not generate any syncytia, and ATL-056i cells, which generated few syncytia under the present coculture conditions (data not shown). The superiority of ATL-040 cells in HTLV-1 transmission may be ascribed to high levels of expression of both envelope gp46 antigen on the cell surface and intracellular Tax antigen (Fig. 1A). Based on these data, we chose ATL-040 cells for *in vivo* infection of CMs with HTLV-1.

### Analysis of CMs directly infected with patient-derived HTLV-1-producing cells.

**FIG 2:**
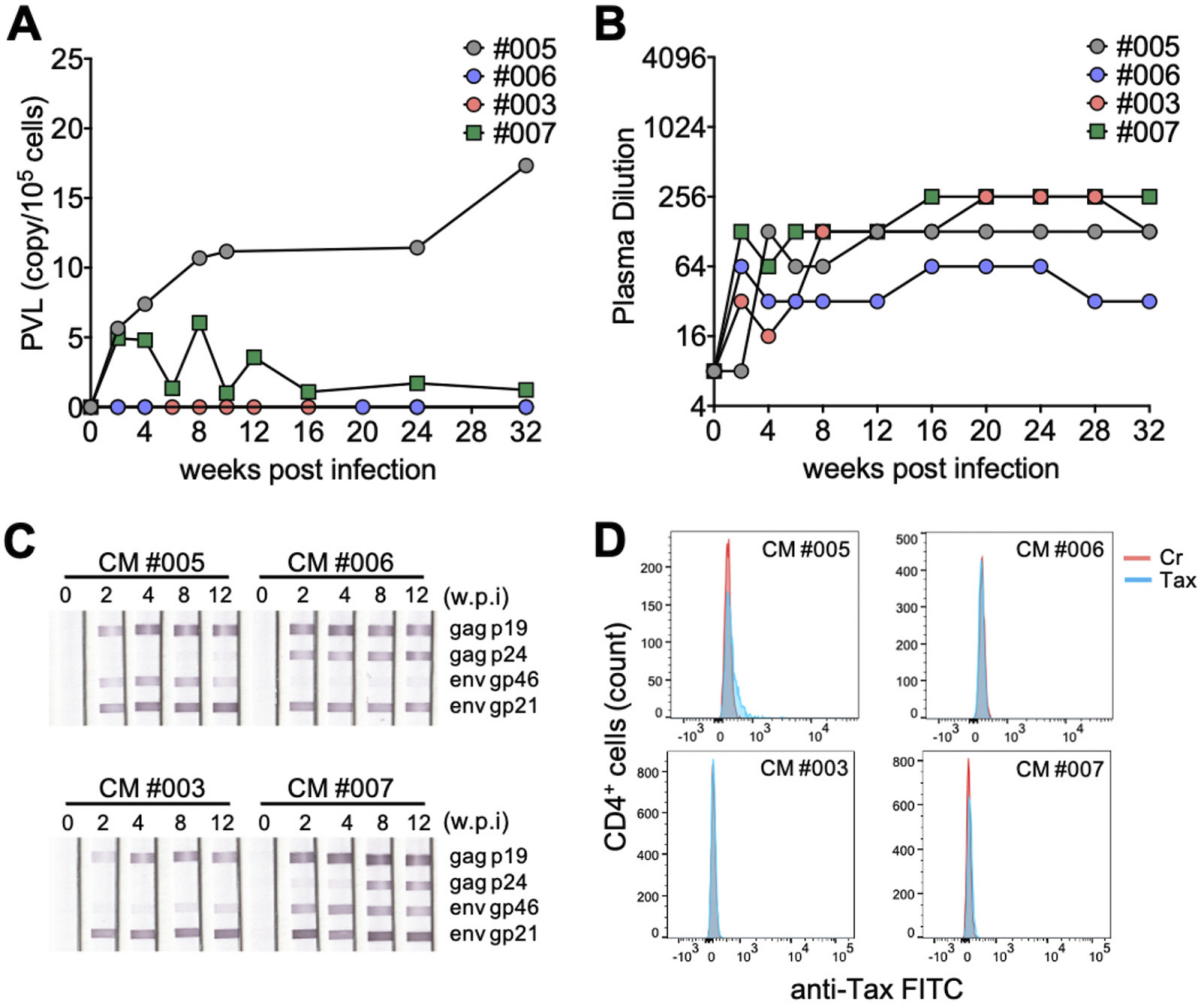
Determination of viral infection in CMs intravenously injected with HTLV-1-infected cells. Two CMs, 005 and 007, were intravenously inoculated with 1 × 10^8^ ATL-040 cells, CM 006 was intravenously inoculated with 1 × 10^8^ ATL-056i cells, and CM 003 was intravenously inoculated with 1 × 10^7^ ATL-040 cells. (A) Proviral load (PVL) in PBMCs (copy/10e5 cells). Provirus was monitored for CMs intravenously injected with HTLV-1-infected cells, and the proviral load (PVL) could be monitored in CMs injected with 1 × 10^8^ ATL-040 cells (005 and 007). (B) The anti-HTLV-1 Ab titer in plasma was determined by the particle agglutination assay. Seroconversion to anti-HTLV-1 Ab was observed in all HTLV-1-infected animals. (C) Line blot analysis using 1:100-diluted plasma samples to detect HTLV-1 antigen-specific Abs. Anti-Env Ab was substantially induced in CMs injected with 1 × 10^8^ ATL-040 cells (CMs 005 and 007). w.p.i., weeks postinfection. (D) Viral protein expression in cells from HTLV-1-infected CMs. Blood samples were cultured overnight in RPMI medium supplemented with 20 U/mL IL-2 and 100 ng/mL IL-4 for samples obtained from CM 005 and CM 006 at 4 weeks and with 20 U/mL IL-2 and 10 μg/mL PHA for samples obtained from CM 003 and CM 007 at 52 weeks. The red lines indicate the histograms of negative-control cells, and the blue lines indicate the histograms of Tax-stained cells.

Three adult female cynomolgus macaques were intravenously injected with 1 × 10^8^ live ATL-040 cells, with CM 005 and CM 007 receiving 1 × 10^8^ cells and CM 003 receiving 1 × 10^7^ cells (Table 1). For comparison, live 1 × 10^8^ ATL-056i cells were injected into CM 006. As determined by quantitation of the proviral loads (PVLs) by semiquantified nested real-time PCR (Fig. 2A), proviral DNA was continuously detected from CM 005 (5 to 10 copies/10^5^ cells) and CM 007 (1 to 5 copies/10^5^ cells), which were injected with 1 × 10^8^ ATL-040 cells, suggesting that 1 × 10^8^ ATL-040 cells let CMs become HTLV-1 carriers. In contrast, the detection of PVLs was negative in CM 003, which was injected with 1 × 10^7^ ATL-040 cells, and CM 006, which was injected with 1 × 10^8^ ATL-056i cells. All of the CMs had seroconverted at 2 weeks after infection, and the levels of anti-HTLV-1 antibody (Ab) titers, measured by particle agglutination assay in plasma samples, were maintained thereafter (Fig. 2B). The CMs injected with ATL-040 cells (005, 003, and 007) showed higher anti-HTLV-1 Ab titers in plasma samples than CM 006, injected with ATL-056i cells. Antigen-specific Abs were confirmed by Western blotting analysis. CMs injected with 1 × 10^8^ ATL-040 cells produced Abs to both viral structural protein Gag and Env (gp46 and gp21), whereas the CM injected with ATL-056i cells (006) and the CM injected with 1/10 the amount of ATL-040 cells (003) showed anti-Gag Ab but weakly induced anti-gp46 Ab (Fig. 2C). Viral-protein-expressing T cells were determined by culturing blood samples overnight in a culture medium supplemented with cytokines for stimulation. Tax-expressing CD4^+^ T cells were detected by FCM in cultured blood samples from CM 005 and CM 007, inoculated with 1 × 10^8^ ATL-040 cells, but not from CM 006, injected with ATL-056i cells, and CM 003, inoculated with 1 × 10^7^ ATL-040 cells (Fig. 2D). Since ATL-040 is a CD3^−^ CD4^−^ B cell line, the Tax-expressing CD4^+^ T cells detected came from HTLV-1-infected monkey cells. These findings indicate that HTLV-1 infection can be established in CMs by direct injection of an HTLV-1-producing cell line, ATL-040, that has a higher transforming ability than MT-2 cells.

**TABLE 1:** Monkey list with experimental design

Monkey	Sex	Age (yr)	HTLV-1-producing cell line administered	No. of cells injected	Route
005	F	7	ATL-040	1 × 10^8^	Intravenous
006	F	7	ATL-056i	1 × 10^8^	Intravenous
003	F	7	ATL-040	1 × 10^7^	Intravenous
007	F	7	ATL-040	1 × 10^8^	Intravenous
011	M	9	ATL-040	1 × 10^8^	Spinal cavity
016	M	3	ATL-040	1 × 10^8^	Spinal cavity
017	F	6	ATL-040	1 × 10^8^	Spinal cavity
014	M	3	ATL-040 after CD8 depletion	1 × 10^8^	Intravenous
015	F	9	ATL-040 after CD8 depletion	1 × 10^8^	Intravenous
018	F	11	ATL-040 after CD8 depletion (225R1)	1 × 10^8^	Intravenous

### Distribution of HTLV-1-infected cells in HTLV-1-infected CMs.

**FIG 3:**
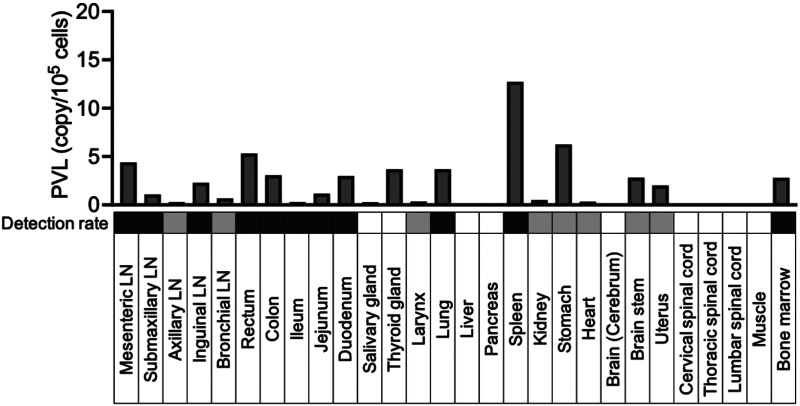
Detection of proviral DNA in indicated tissues and organs in an HTLV-1-chronically infected CM. CM 005, which was intravenously injected with ATL-040 cells, was sacrificed at 101 weeks postinfection, and the PVLs in different tissue samples were calculated by PCR. Experiments were performed in duplicate, and DNAs from tissue samples were analyzed in at least two independent experiments. The gray scale shows the detection rate of proviral DNA (black, more than 50%; gray, less than 50%). LN, lymph nodes.

Since CM 005, which was injected with 1 × 10^8^ ATL-040 cells, showed a slightly higher PVL than CM 007, CM 005 was sacrificed at 101 weeks postinfection and the distribution of HTLV-1 provirus in several tissues and organs was determined by PCR, using the same method as was used for the measurement of PVL. Due to the detection limit suggesting small amounts of proviral DNA in tissues and organs, samples were tested by PCR in duplicate in at least two independent experiments. As shown by the results in Fig. 3, proviral DNA was detected in bone marrow, major lymph nodes, intestine, spleen, and lung with high detection rates and with certain levels of copy numbers. These localizations were similar to the HTLV-1 distribution determined in HTLV-1-infected squirrel monkeys (36) and the simian T-cell leukemia virus (STLV) distribution in Japanese macaques (37). Taken together, the results indicate that sustained PVL and anti-HTLV-1 Ab titers are maintained for more than 100 weeks in CMs that are intravenously injected with 1 × 10^8^ ATL-040 cells. The results also indicate that CMs are highly susceptible to productive HTLV-1 infection and that chronic HTLV-1 infection can be established by direct injection of ATL-040 cells.

### The effect of CD8^+^ T cell depletion on HTLV-1 propagation in HTLV-1-infected CMs.

**FIG 4:**
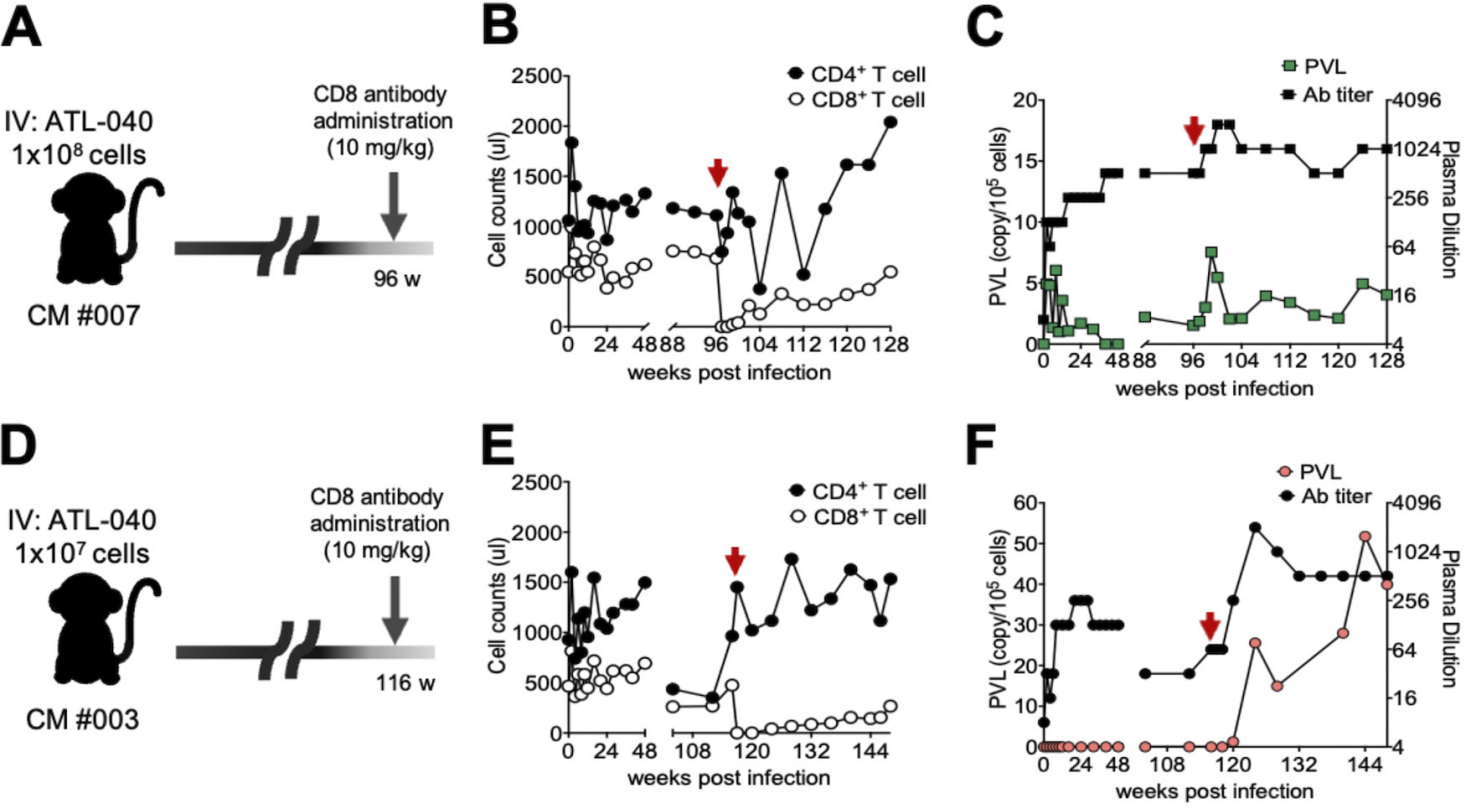
Effects of CD8^+^ T cell depletion on chronically HTLV-1-infected animals. (A) HTLV-1-infected CM 007 was subcutaneously administered an anti-CD8 MAb at 96 w.p.i., weeks postinfection. (B) CD4^+^ (solid) and CD8^+^ (open) T cell counts in CM 007. (C) Changes in the PVLs in PBMCs (green) and anti-HTLV-1 Abs in plasma (black) before and after CD8^+^ T cell depletion. (D) Low-dose HTLV-1-infected CM 003 was subcutaneously administered an anti-CD8 Ab at 116 w.p.i. (E) CD4^+^ (solid) and CD8^+^ (open) T cell counts in CM 003. (F) The PVLs in PBMCs became detectable (red) and anti-HTLV-1 Ab levels increased (black) after CD8^+^ T cell depletion. (C, F) The red arrows indicate the times of anti-CD8 Ab administration.

CD8^+^ T cells play a critical role in the control of viral infections, such as human immunodeficiency virus (HIV) infection (38–40). To determine the effects of CD8^+^ T cells on chronic HTLV-1 infection, HTLV-1-infected CMs were subcutaneously administered 10 mg/kg of body weight of an anti-CD8 monoclonal antibody (MAb) (Fig. 4A). CM 007, with sustained chronic infection, was treated with an anti-CD8 MAb at 96 weeks after infection. CD8^+^ T cells were immediately depleted after administration and gradually recovered after 1 month (Fig. 4B). The PVLs and Ab levels in plasma increased in parallel after the depletion of CD8^+^ T cells and then returned to the levels before depletion in CM 007 (Fig. 4C). The PVL could not be monitored in CM 003, which was injected with 1 × 10^7^ ATL-040 cells, despite the fact that anti-HTLV-1 Abs were maintained for more than 2 years. Interestingly, when CM 003 was administered the anti-CD8 MAb at 116 weeks postinfection (Fig. 4D and E), the PVL not only became measurable but also showed a high load (Fig. 4F). CD3^+^ CD4^+^ cells and CD4^−^ CD20^+^ cells were isolated from PBMCs at 144 weeks, and proviral DNA was detected by digital PCR. Proviral DNA was enriched in CD3^+^ CD4^+^ cells (154/10^5^ cells) but was not detected in CD4^−^ CD20^+^ cells. The plasma Ab titer was also dramatically increased 2 weeks after administration of the anti-CD8 MAb (Fig. 4F). These results indicate that latent HTLV-1 infection was established in CM 003, which was injected with 1 × 10^7^ ATL-040 cells, and that CD8^+^ T cells contributed to the suppression of HTLV-1 propagation *in vivo*. The results suggest that CD8^+^ T cells play a critical role in virus control in the present model.

### Impact of immune escape at the time of HTLV-1 infection.

**FIG 5:**
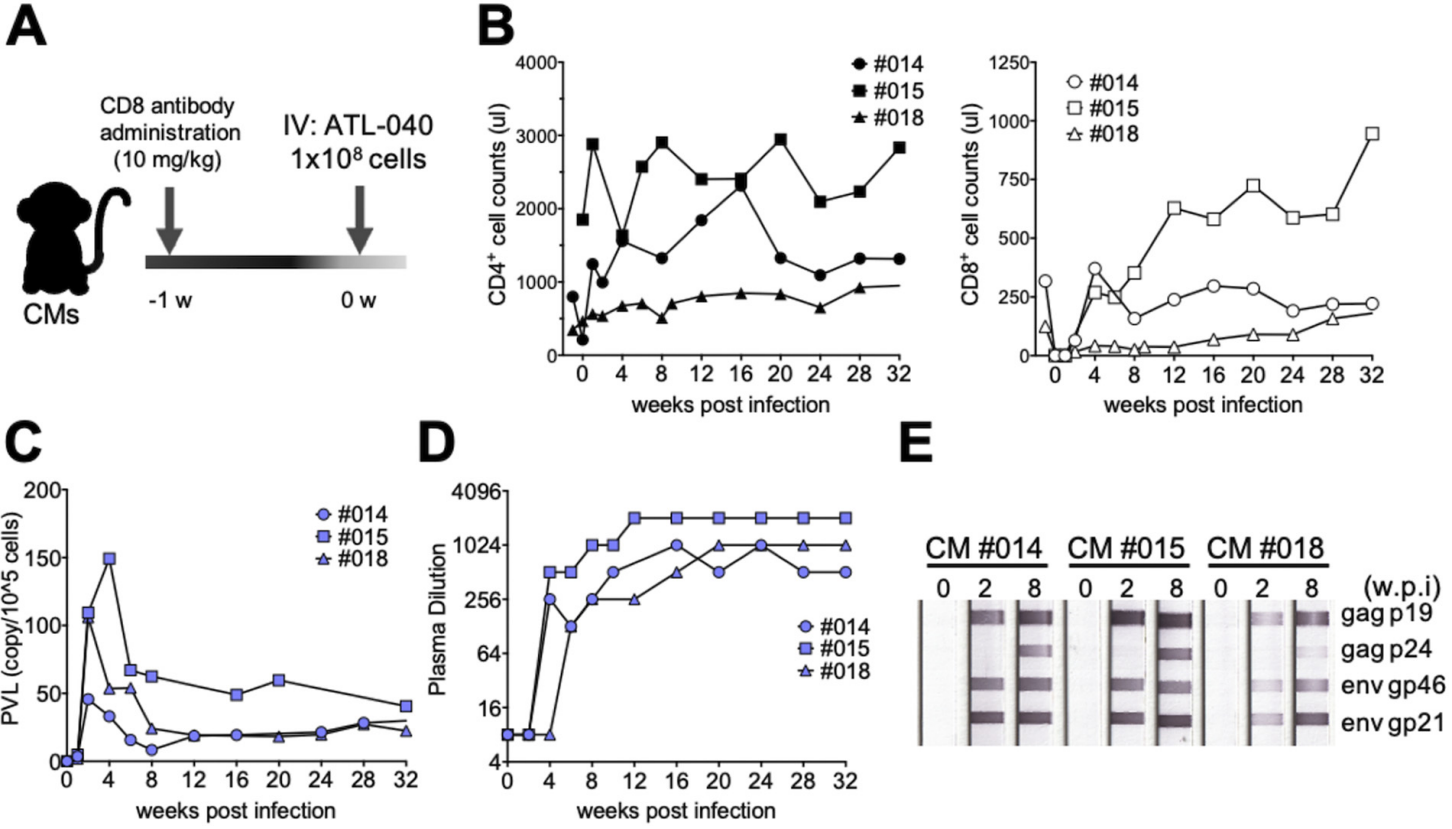
Effects of CD8^+^ T cells on HTLV-1 infection at the time of infection. (A) CD8^+^ T cells were depleted 1 week before infection in three CMs. (B) CD4^+^ (left) and CD8^+^ (right) T cell counts in CMs administered anti-CD8 MAb. (C, D) The PVLs in PBMCs (C) and anti-HTLV-1 Abs in plasma (D) were monitored in animals administered anti-CD8 MAb. (E) Levels of antigen-specific Abs were determined.

The injection of ATL-040 cells resulted in the establishment of HTLV-1 infection in CMs with a 100% success rate. However, the PVLs were as low as those in human HTLV-1 carriers. One of the possible causes may be that ATL-040 cells are xenogeneic. ATL-040 cells inoculated into CMs may be attacked by xenoimmunity mechanisms. To avoid such immediate elimination by the host immune response, CMs were administered an anti-CD8 MAb 1 week before infection (Fig. 5A). Depletion of CD8^+^ T cells at the time of infection was confirmed by FCM (Fig. 5B). Intravenous injection of 1 × 10^8^ ATL-040 cells into CD8^+^ T cell-depleted CMs led to significantly high PVLs compared to those in untreated CMs 005 and 007 (Fig. 2 and 5C). The anti-HTLV-1 Ab titers of the CD8^+^ cell-depleted CMs were slightly higher than those in CMs that were not administered the anti-CD8 MAb (Fig. 5D and E). These results were consistent with those shown in Fig. 4 and supported that CD8^+^ T cells also contributed to the suppression of HTLV-1 propagation at an early stage of HTLV-1 infection.

**FIG 6:**
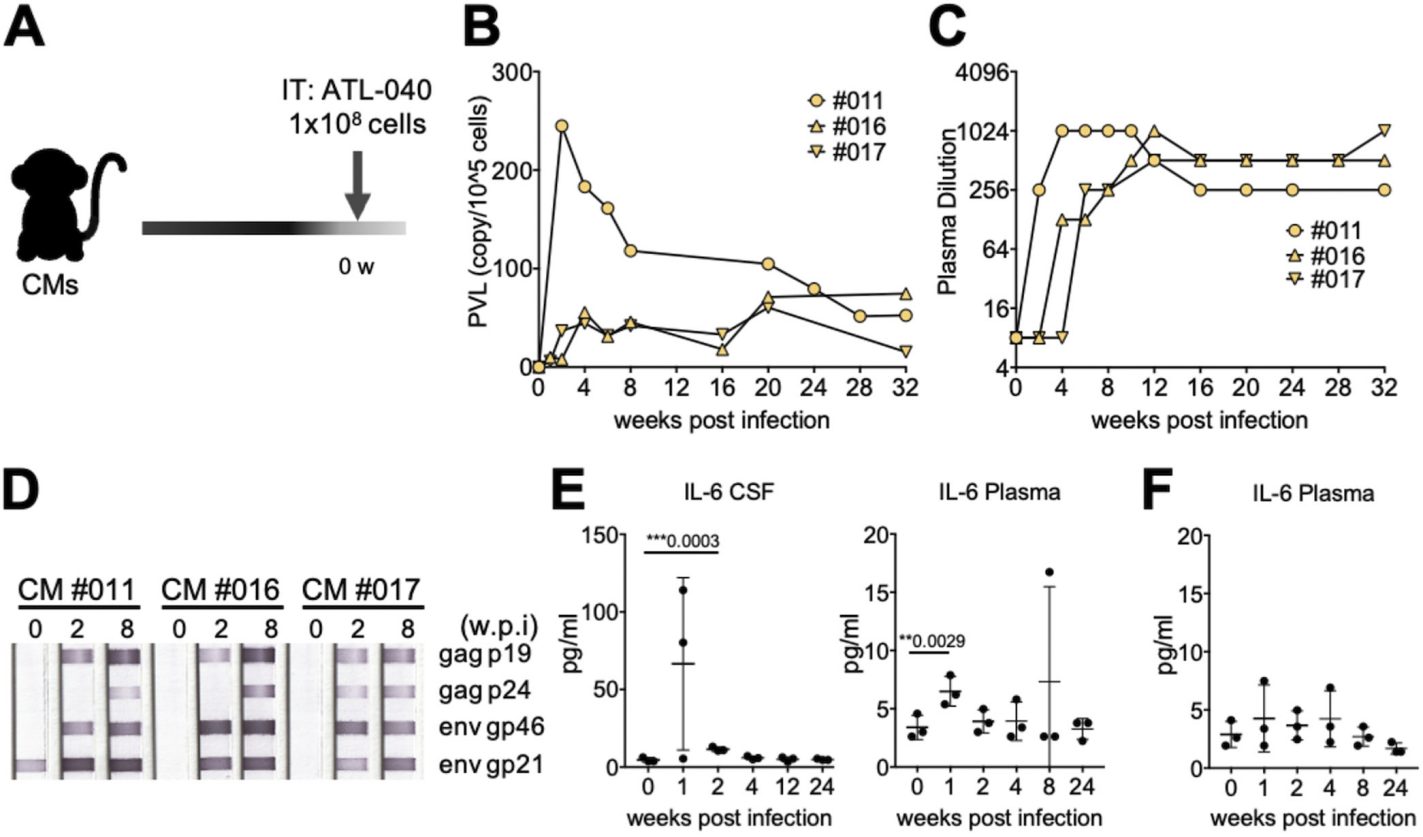
Analysis of HTLV-1 infection in CMs intrathecally injected with ATL-040 cells. IT, intrathecal. (A) Three CMs were infected with HTLV-1 by intraspinal injection. (B, C) The PVLs in PBMCs (B) and anti-HTLV-1 Abs in plasma (C) were monitored after intrathecal infection. (D) Antigen-specific Abs were determined. (E) The levels of 29 cytokines and chemokines in CSF and in plasma were analyzed. IL-6 was increased transiently in both CSF (left) and plasma (right) after infection. (F) Levels of IL-6 in plasma in CMs that were preadministered an anti-CD8 Ab. Statistical analyses between the indicated time points were analyzed by the two-tailed paired *t* test. Bars and whiskers show mean values ± standard deviations. **, *P* < 0.01; ***, *P* < 0.001.

Next, we examined the effect of intrathecal administration of HTLV-1, because there are no immune cells in cerebrospinal fluid (CSF) under normal conditions (Fig. 6A). Injection of the same number of ATL-040 cells (1 × 10^8^ cells) into the spinal cavity of CMs resulted in substantially high PVLs in PBMCs and slightly higher anti-HTLV-1 Ab titers that were similar to those seen in CMs that were preadministered the anti-CD8 MAb (Fig. 6B to D). Curiously, an anti-gp21 Ab band was detected in CM 011 before HTLV-1 inoculation (Fig. 6D). This anti-gp21 Ab signal was not absorbed in an absorption test (data not shown). Since plasma samples were negative in a particle agglutination test of anti-HTLV-1 Ab (Fig. 6C) and anti-STLV Ab, we concluded that this reaction was nonspecific. Interestingly, the proinflammatory cytokine IL-6 was transiently but significantly elevated at 2 weeks postinfection in CSF and at 1 week in plasma samples from intrathecally infected CMs (Fig. 6E) but not in samples from CMs that were preadministered the anti-CD8 MAb (Fig. 6F). Although HAM/TSP-related symptoms and other clinical symptoms were not observed during the experimental period, the present study suggested that using CMs with HTLV-1 infection with an immunological approach might be a useful animal model to study HTLV-1 infection and host defense mechanisms and its associated disease development. An HTLV-1 CM model would provide great advantages for the development of prophylactic vaccine candidates and novel therapies.

## DISCUSSION

The use of an animal model of HTLV-1 infection is important for elucidation of the pathophysiology and for the development of preventive and therapeutic agents. Rats have mainly been used as an animal model for HTLV-1 infection; however, the divergence of rats from humans is large and the reflection of human pathology is not sufficient (41, 42). Since HTLV-1-related diseases require a long period of chronic infection, animals with a long life span, such as NHPs, would be suitable. NHPs are close to humans and are considered to be extremely suitable as animal models for conducting HTLV-1 studies if a stable infection model can be established. In addition to the immunological and anatomical similarities between humans and NHPs, we consider that the CM is an ideal animal for HTLV-1 research because, unlike other macaques, the CM is not a seasonal breeding animal and it has a regular menstrual period. Since mother-to-child transmission through breastfeeding is one of the major HTLV-1 infection routes, the animals are applicable for simulating vertical transmission. Also, the body size of CMs is smaller than those of rhesus and Japanese macaques, and CMs are therefore easier to handle and are useful for pharmaceutical evaluation because they allow for saving of drug doses. In the present study, we established a CM model of HTLV-1 infection by direct injection of an HTLV-1-producing cell line, ATL-040, derived from an ATL patient, and we analyzed this model in detail.

**FIG 7:**
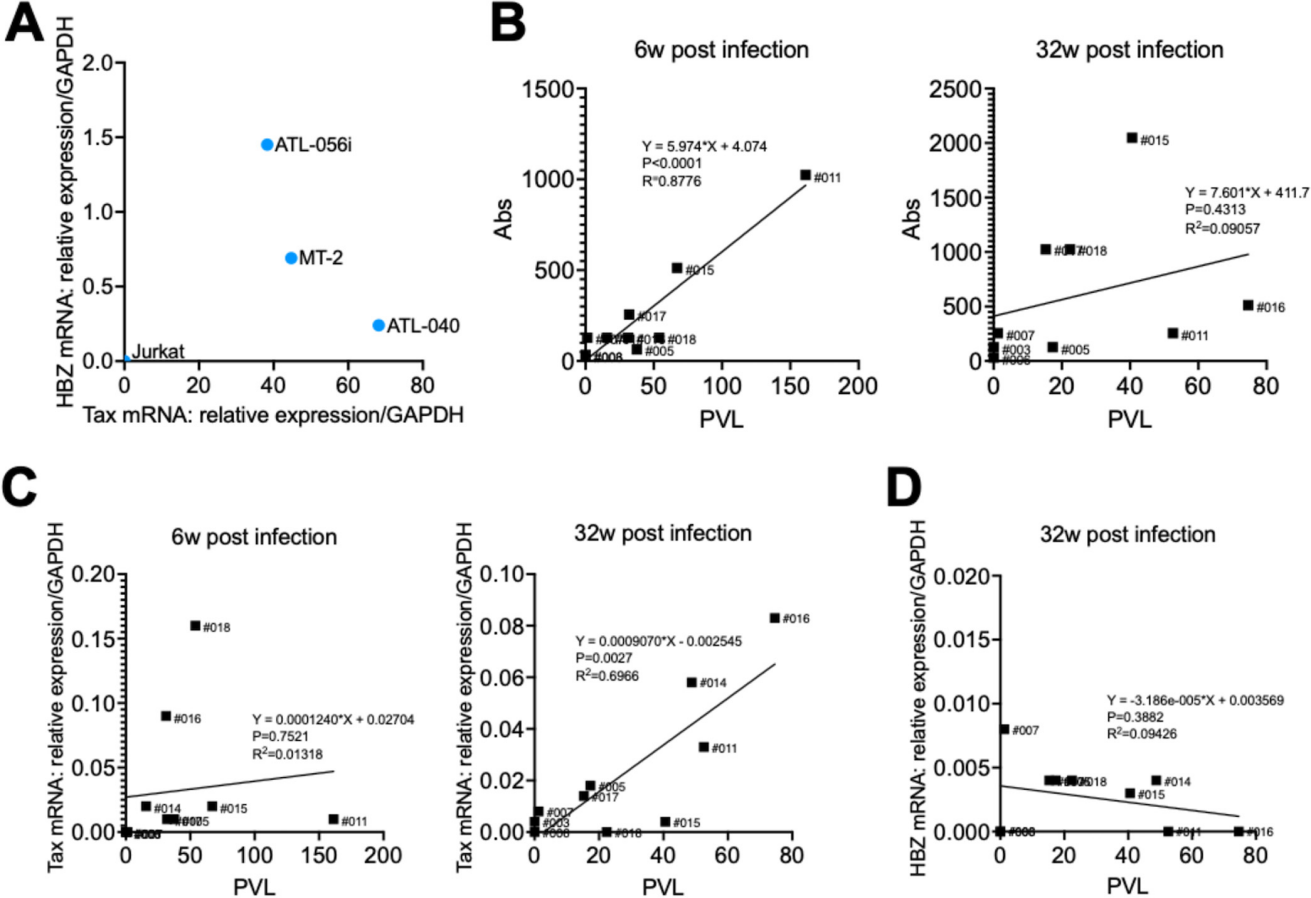
Comparisons of the Tax and HBZ mRNA levels and of the PVLs and anti-HTLV-1 Ab titers among HTLV-1-infected CMs. (A) Tax and HBZ mRNA levels of HTLV-1-producing cell lines. (B) A positive correlation was observed between the PVLs and anti-HTLV-1 Ab titers at 6 weeks (left) but not at 32 weeks (right). (C) A positive correlation was observed between the Tax mRNA expression levels and PVLs at 32 weeks (right) but not at 6 weeks (left). (D) HBZ mRNA was expressed in some HTLV-1 CMs, but no significant correlation was observed between the HBZ mRNA expression levels and PVLs at 32 weeks.

The selection of cells producing HTLV-1 is important for the establishment of infection, since infection has not been established in NHPs by direct inoculation of traditionally used cells (31, 33). The ATL-040 cell line, which is an IL-2-independent B cell line isolated from an ATL patient, showed a more rapid and stronger cytopathic effect (CPE) than other cells (Fig. 1D). In addition, Tax mRNA expression levels were also higher in ATL-040 cells than in MT-2 and ATL-056i cells (Fig. 7A). The CPE results indicate that the infectivity of ATL-040 cells is high, and the higher expression of Tax indicates that initial HTLV-1 infection could be established by direct inoculation of cells into CMs.

**FIG 8:**
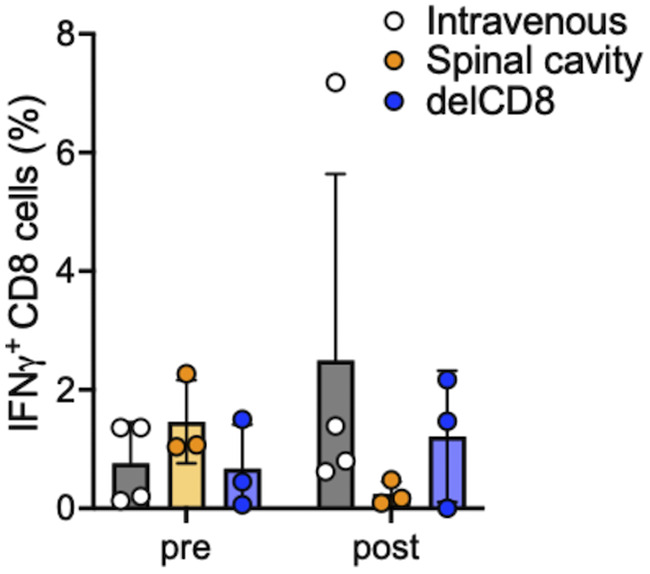
Antigen-nonspecific CD8^+^ T cell response. Percentages of CD8 T cells in PBMCs producing IFN-γ. The CD8 response to SEB was determined preinfection (pre) and at 9 to 12 weeks postinfection (post). delCD8, CD8 depleted before infection. Error bars show standard deviations.

HTLV-1 infection is mostly asymptomatic, and few people with HTLV-1 infection develop ATL or HAM/TSP. Some cross-sectional studies have shown a relationship between the onset of ATL and HAM/TSP and the levels of PVL. It has been shown that a high PVL increases the risk of development of HTLV-1-associated complications (43, 44) and that a high PVL may prospectively predict the risk of ATL (45) and HAM/TSP (46). CMs injected with HTLV-1-infected ATL-040 cells showed persistent infection with a low PVL (Fig. 2). The PVL produced by intravenous injection of HTLV-1-infected cells seems to be low compared to those in Japanese macaques naturally infected with STLV-1 and some asymptomatic carriers (47, 48). On the other hand, some asymptomatic carriers also have low levels of PVL (49), similar to an intravenous injection CM model. There is a limit for obtaining human specimens and for studying HTLV-1 proliferation in asymptomatic carriers, and the HTLV-1-infected CM model is therefore suitable for studying the pathogenesis of carriers before the development of ATL and HAM/TSP. PVL was detected stably and at high levels in the lymph nodes, spleen, and intestine, which are potential sources of the HTLV-1 infection reservoir, in CM 005 (Fig. 3), and these results were consistent with the results of previous studies using mice (50), squirrel monkeys (36), and STLV-1-infected Japanese macaques (37). HTLV-1 proviral DNA was also detected in relevant organs, including the genital organs, as wells as the heart and kidney and other major organs. Although HTLV-1 is transmitted vertically from mother to child through breastmilk, the possibilities of intrauterine infection and infection during delivery cannot be excluded (47). This intravenous injection model will be useful for evaluating the efficacy of prevention procedures. On the other hand, CMs from which CD8^+^ T cells had been eliminated prior to infection showed high PVLs and HTLV-1-specific Ab responses (Fig. 5C and D). Also, HAM/TSP is an immune-mediated inflammatory disease characterized by demyelination of motor neurons in the spinal cord, and we therefore investigated whether direct injection of HTLV-1-producing cells into the spinal cavity would trigger the development of HAM/TSP. CMs with intraspinal injection of HTLV-1-infected cells also showed high PVLs and Ab responses (Fig. 6B and C). A positive correlation between PVLs and anti-HTLV-1 Ab titers was observed at the early phase of infection among the HTLV-1-infected CMs tested in this study, but the positive correlation was not significant at the chronic phase (Fig. 7B). On the other hand, Tax mRNA expression was positively correlated at the chronic phase (Fig. 7C). Tax expression was high and HTLV-1 bZIP factor (HBZ) expression was low in ATL-040 cells compared to their expression levels in other HTLV-1-producing cell lines (Fig. 7A). HBZ mRNA was not detected in any of the CMs’ PBMCs at 6 weeks, but HBZ mRNA was detectable in some CMs at 32 weeks (Fig. 7D). Since both Tax and HBZ are involved in viral propagation and pathogenesis (51–53), prolonged monitoring of the expression of these genes and the accumulation of data might be important for understanding the variations in pathological and virological conditions of HTLV-1 infection. Although anti-HTLV-1 Ab was negative in CSF and the levels of the inflammation marker CXCL10 (IP-10), which is elevated in HAM/TSP patients (48, 49), were not changed before and after infection in intrathecally inoculated CMs (data not shown), immune regulation, including CD8^+^ T cell depletion and use of the immune evasion route at the time of infection, caused higher PVLs in CMs. HTLV-1-infected human cell lines are usually excluded as heterologous animal cells in CMs, but it seems that it took time to eliminate the HTLV-1-infected human cells due to the removal of CD8^+^ T cells and the administration of infected cells into the spinal cord without leukocytes. In addition, when we examined the antigen-nonspecific CD8^+^ T cell response, interferon-gamma positive (IFN-γ^+^) CD8^+^ T cells were increased in intravenously inoculated CMs and in CMs preadministered an anti-CD8 MAb after the inoculation of cells compared to the levels before infection (Fig. 8). In contrast, IFN-γ^+^ CD8^+^ T cells were not increased in intrathecally inoculated CMs. Although future analyses are needed, including analysis of antigen-specific T cell responses, these results suggest that intrathecal inoculation can establish the HTLV-1 infection in CMs sneakily, without an immune response of CD8^+^ T cells, and this might be the reason why these CMs showed a high PVL. This procedure for inducing a high PVL in CMs may be useful for understanding host viral-control mechanisms and the sequential development of disease. Since HAM/TSP-related symptoms and other clinical symptoms were not observed during the experimental period, it is therefore necessary to observe CMs showing a high PVL for a longer period of time. Interestingly, the dependence on CD8^+^ T cells to control virus infection was varied in CMs that were chronically infected with HTLV-1. The PVL was increased after depletion of CD8^+^ T cells in CM 007, and the PVL returned to the level before depletion of CD8^+^ T cells after the CD8^+^ T cells reached the pretreatment level. On the other hand, in CM 003, which was without a detectable PVL before depletion of CD8^+^ T cells, the PVL became not only detectable but also continuously high after the level of CD8^+^ T cells had recovered (Fig. 4F). Since HTLV-1 infection causes several diseases and some carriers develop disease after surgery with immunosuppressive conditions, the host defense mechanisms against HTLV-1 may vary. Extensive studies on immunoregulation in the CM model of chronic HTLV-1 infection and the accumulation of data using the CM model might lead to an understanding of the host defense mechanisms against HTLV-1 infection and might contribute to the prevention of disease onset.

The CM model of HTLV-1 infection established in this study should contribute to the elucidation of the complicated immunopathology of HTLV-1 infection and to the development of vaccines and therapeutic agents.

## MATERIALS AND METHODS

### Cells.

MT-2 cells and Jurkat cells were maintained in RPMI 1640 medium with fetal bovine serum (FBS) (10%, vol/vol), glutamine (2 nM), penicillin (100 U/mL), and streptomycin (100 μg/mL). The HTLV-1-producing cells lines tested, ATL-056i and ATL-040, were derived from ATL patients (34, 50). ATL-040 cells, which are IL-2-independent B cells, were maintained in RPMI 1640 medium with FBS (12%, vol/vol), glutamine (2 nM), penicillin (100 U/mL), and streptomycin. ATL-056i cells, which are IL-2-dependent T cells, were maintained in RPMI 1640 medium with FBS (10%, vol/vol), glutamine (2 nM), penicillin (100 U/mL), streptomycin, and IL-2 (20 U/mL) (Shionogi). All cells were cultured at 37°C in a humidified incubator with 5% CO_2_.

### Animals.

Cynomolgus macaques (CMs) that were reared under specific-pathogen-free (SPF) conditions at Tsukuba Primate Research Center, National Institutes of Biomedical Innovation, Health and Nutrition (NIBIOHN, Ibaraki, Japan), were used in this study after approval by the Committee on Ethics of Animal Experiments of NIBIOHN in accordance with the guidelines for animal experiments at NIBIOHN. All of the CMs used in this study were negative for STLV, simian immunodeficiency virus (SIV), simian retrovirus (SRV), simian varicella virus (SVV), and B virus. The animals were housed individually.

### *In vitro* transformation assay.

Human PBMCs from normal donors and PBMCs derived from a cynomolgus monkey were used as indicator cells. These indicator cells (2 × 10^6^ cells) were cocultured with mitomycin C (MMC)-treated HTLV-1-producing cells (2 × 10^5^ cells) in a volume of 0.5 mL/well in a 48-well plate (Falcon) in RPMI 1640 medium supplemented with 10% FBS, 100 U/mL penicillin, 100 μg/mL streptomycin, 100 U/mL human IL-2 (Miltenyi), and 10 μg/mL phytohemagglutinin-P (PHA; Sigma) at 37°C for 2 weeks. The medium was changed for fresh medium without PHA two times per week. Then, the cells were stained for cell surface CD4 and HTLV-1 Tax antigen as reported previously (51). A syncytium formation assay was performed by coculturing HTLV-1-producing cells and HTLV-1-negative Jurkat cells. Equal numbers of cells were cultivated for the indicated time periods, and then CPE was confirmed by microscopy.

### HTLV-1 infection.

CMs were intravenously infected with 1 × 10^7^ to 1 × 10^8^ HTLV-1-producing ATL-040 cells or ATL-056i cells derived from ATL patients in 10 mL phosphate-buffered saline (PBS). CMs 003 and 007 were administered 10 mg/kg anti-CD8 Ab (MT807R1) (NIH Nonhuman Primate Reagent Resource) subcutaneously at 116 weeks and 96 weeks postinfection, respectively. Three CMs were subcutaneously administered 10 mg/kg anti-CD8 Ab (CMs 014 and 015 [MT807R1] and CM 018 [225R1]) (NIH Nonhuman Primate Reagent Resource) and then intravenously injected with 1 × 10^8^ ATL-040 cells in 10 mL PBS 1 week later. CMs 011, 016, and 017 were intrathecally injected with 1 × 10^8^ ATL-040 cells in 1 mL PBS using an epidural kit (B. Braun Aesculap).

### PBMC isolation and DNA extraction.

Human PBMCs were isolated by density centrifugation using lymphocyte separation solution (nacalai tesque, Inc., Kyoto, Japan). Monkey PBMCs were isolated by density centrifugation using Lympholyte mammal cell separation medium (Cedarlane). DNA was extracted from PBMCs using the MagNA Pure compact NA isolation kit I (Roche Diagnostics) or MagDEA Dx SV (Precision System Science Co., Ltd.). Tissue samples were homogenized by using MagNA Lyser green beads with the MagNA Lyser instrument, and then sample lysates were incubated with 2 mg/mL RNase A (Qiagen) for 15 min at 65°C and DNA was extracted as described above.

### Quantification of HTLV-1 proviral loads.

The HTLV-1 proviral loads in PBMCs and several tissues were semiquantified by nested PCR. For each sample, 1,000 ng of genomic DNA extracted as described above was used as the template for the first PCR. The first PCR was performed with Hot Start *Ex Taq* (TaKaRa) according to the manufacturer’s instructions. In brief, 50 μL of a total PCR mixture containing HTLV-1 pX outer primer set pX1 (5′-CCCACTTCCCAGGGTTTGGACAGAGTCTTC-3′) and pX4 (5′-GGGGAAGGAGGGGAGTCGAGGGATAAGGAA-3′) with a concentration of 0.25 μM each was used to perform 20 cycles of touchdown PCR with an annealing temperature from 65°C to 60°C. Then 5 μL of the first PCR’s product was subjected to the second PCR, a quantitative real-time PCR, using TaqMan fast advanced master mix (Thermo Fisher Scientific) according to the manufacturer’s instructions on a LightCycler 480 II (Roche Diagnostics). In the second PCR, the inner primer set pX S (5′-CGGATACCCAGTCTACGTGTT-3′) and pX AS (5′-CAGTAGGGCGTGACGATGTA-3′) with pX2 probe (6-carboxyfluorescein [FAM]-5′-CTGTGTACAAGGCGACTGGTGCC-3′-Black Hole Quencher 1 [BHQ1]) was used (52, 53). The input DNA cell equivalent value was measured in the same sample by the abundance of recombination-activating gene 1 (Rag-1 gene) using the following primer and probe set: Rag-1 Fwd (5′-CCCACCTTGGGACTCAGTTCT-3′), Rag-1 Rev (5′-CACCCGGAACAGCTTAAATTTC-3′), and mfRAG-1 probe (HEX-5′- CCCCAGATGAAATTCAGCACCCATA-3′-BHQ1). The reaction conditions of the second PCR were 95°C for 10 min (activation of the polymerase) and 45 cycles of 15 s at 95°C (denaturing) followed by 60 s at 60°C (annealing and extension). All experiments were performed in duplicate, and DNAs from tissue samples were analyzed in at least two independent experiments. PBMCs from CM 003 were subjected to cell sorting. Cells were stained using the following monoclonal antibodies: anti-CD3 Ab (clone SP34-2, conjugated with peridinin chlorophyll protein [PerCP]; BD), anti-CD4 Ab (clone L200, conjugated with phycoerythrin [PE]; BD), anti-CD8 Ab (clone SK1, conjugated with allophycocyanin [APC]; BioLegend), and anti-CD20 (clone 2H7, conjugated with fluorescein isothiocyanate [FITC]; BD). CD3^+^ CD4^+^ CD8^−^ cells and CD4^−^ CD20^+^ cells were sorted by using the FACSMelody (BD). DNAs were extracted using the QIAamp DNA minikit (Qiagen) according to the manufacturer’s instructions. DNAs were subjected to crystal digital PCR (dPCR) analysis (Naica system) using a sapphire tip (Stilla) according to the manufacturer’s instructions on a Naica Geode (Stilla). dPCR was performed using Perfecta multiplex qScript ToughMix (Quantabio) with the primer and probe set pX S, pX AS, and pX2 probe (HEX-5′-CTGTGTACAAGGCGACTGGTGCC-3′-BHQ1). For the reference gene, the following primer and probe set was used: Rag-1 Fwd, Rag-1 Rev, and mfRAG-1 probe (FAM-5′-CCCCAGATGAAATTCAGCACCCATA-3′-BHQ1). The reaction conditions of dPCR were 40°C for 12 min (partition), 95°C for 10 min (initial denaturation), and 45 cycles of 30 s at 95°C (denaturing) followed by 30 s at 60°C (annealing and extension), and then the sapphire tips were scanned and analyzed in a Naica Prism3.

### Detection of anti-HTLV-1 antibody.

The anti-HTLV-1 Ab titers in plasma and CSF were determined by the particle agglutination method using Serodia HTLV-1 (Fuji Rebio, Inc.). Antigen-specific antibodies anti-Gag Ab and anti-Env Ab in plasma samples at 1:100 dilutions were confirmed by the Inno-LIA HTLV I/II Score line blot assay (Fuji Rebio, Inc.).

### Detection of HTLV-1 viral-protein-expressing cells.

Whole-blood samples were cultured overnight in RPMI medium supplemented with 20 U/mL IL-2 (Shionogi) and 100 ng/mL IL-4 (R&D Systems) for samples from CM 005 and CM 006 and with 20 U/mL IL-2 and 10 μg/mL phytohemagglutinin-P (PHA; Sigma) for samples from CM 003 and CM 007. Then, Tax antigen-expressing cells were analyzed by FCM. Whole-blood samples obtained at 4 weeks for CM 005 and CM 006 and at 52 weeks for CM 003 and CM 007 were used.

### Cytokine and chemokine profiles.

The profiles of cytokines and chemokines in CSF and plasma were analyzed using a monkey cytokine magnetic 29-plex panel for the Luminex platform (Invitrogen, Thermo Fisher Scientific) according to the manufacturer’s protocol. Signals were detected and analyzed using the Bio-Plex 200 system (Bio-Rad Laboratories) with Bio-Plex Manager 6.0 software (Bio-Rad Laboratories).

### Flow cytometry analysis.

ATL-040 and ATL-056i cells were preincubated with 2 mg/mL normal human IgG for 10 min and then stained with appropriate combinations of MAbs. For detection of gp46 and Tax, cells were fixed in 1% paraformaldehyde (PFA) and permeabilized in 0.1% saponin, followed by staining with fluorochrome-labeled anti-gp46 (LAT-27) and anti-Tax (Lt-4) MAbs. The following MAbs were used: anti-CD8-FITC MAb (clone OKT8; in-house labeled), anti-CD4-PE MAb (clone OKT4; Biolegend), anti-CD25-PE-cyanine 7 (PC7) MAb (clone BC6; Biolegend), anti-CD3-HyLite Fluor 647 (HF647) MAb (clone OKT-3, in-house labeled), anti-CD7-PC7 MAb (clone CD7-6B7; Biolegend), anti-CADM-1-PE MAb (clone 3E1; MBL), anti-CD19-FITC and anti-CD20-PE MAbs (Beckman Coulter), and anti-HTLV-1 gp46-FITC (LAT-27) and anti-Tax-HF647 (Lt-4) MAbs (in-house labeled).

To analyze CD4^+^ T cell and CD8^+^ T cell counts, 100 μL of whole blood from each of the CMs was stained using the following monoclonal antibodies: anti-CD3 MAb (clone SP34-2, Alexa Fluor 700 labeled; BD), anti-CD4 MAb (clone L200, conjugated with PerCP; BD), and anti-CD8 MAb (clone DK25, conjugated with APC; Dako). Tax expression was determined using an anti-Tax antibody (clone Lt-4). Cells stained with cell surface makers were permeabilized by BD Cytofix/Cytoperm (BD) according to the manufacturer’s protocol, and then the cells were incubated with a 1:10 dilution of FITC-labeled anti-Tax antibody (clone Lt-4) for 30 min. Negative-control cells were stained with FITC-Lt-4 in the presence of a 1:10 dilution of unlabeled Lt-4. Stained cells were fixed with 1% freshly prepared paraformaldehyde for at least 2 h and then analyzed in a FACSCanto II or LSRFortessa X-20 flow cytometer (BD). Data were analyzed using FlowJo (BD) software.

### Analysis of antigen-nonspecific CD8^+^ T cell responses.

Antigen-nonspecific cellular immune responses were assessed in multiparameter intracellular cytokine staining (ICS) assays. Cryopreserved PBMCs were cultured in RPMI medium with 10% FBS at 37°C overnight before staphylococcal enterotoxin B (SEB) (Sigma) stimulation. Cells were stimulated with SEB at 1 μg/mL for 3 h and incubated for an additional 6 h with GolgiStop at 0.7 μL/mL (BD Biosciences). A fixable-dead-cells staining kit (near-IR; Invitrogen) was used to exclude dead cells from the analysis, and then immunostaining was performed using a Cytofix/Cytoperm kit (BD) and the following monoclonal antibodies: anti-CD3 MAb (clone SP34-2, Alexa Fluor 700 labeled; BD), anti-CD4 MAb (clone OKT4 BV421; BD), anti-CD8 MAb (clone DK25, conjugated with APC; Dako), and anti-IFN-γ MAb (clone 4S.BS, conjugated with PE; BD). Stained cells were fixed with 1% freshly prepared paraformaldehyde for at least 2 h and then analyzed in an LSRFortessa X-20 flow cytometer. Data were analyzed using FlowJo software.

### Analysis of Tax and HBZ mRNA expression.

RNA was extracted from cell line cultures using MagDEA Dx SV RNA (Precision System Science). Cryopreserved PBMCs were cultured in RPMI medium, 10% FBS with 20 U/mL IL-2 for 2 days, and then RNAs were extracted using an RNeasy minikit (Qiagen) according to the manufacturer’s instructions. RNAs were subjected to crystal reverse transcription (RT)-dPCR analysis using a sapphire tip according to the manufacturer’s instructions on a Naica Geode. RT-dPCR was performed using qScript-XLT 1-step RT-quantitative PCR (qPCR) ToughMix (Quantabio) with the following primer and probe sets (54–57): Tax mRNA Fwd (5′-ATCCCGTGGAGACTCCTCAA-3′), Tax mRNA Rev (5′-CCAAACACGTAGACTGGGTATCC-3′), and Tax mRNA probe (HEX-5′-TCCAACACCATGGCCCACTTCCC-3′-BHQ1); HBZ mRNA Fwd (5′-AGAACGCGACTCAACCGG-3′), HBZ mRNA Rev (5′-TGACACAGGCAAGCATCGA-3′), and HBZ mRNA probe (Cy5-TGGATGGCGGCCTCAGGGCT-3′-BHQ2); and glyceraldehyde-3-phosphate dehydrogenase (GAPDH) Fwd (5′-ATGTTCCAGTATGATTCCACCC-3′), GAPDH Rev (5′-CATCGCCCCACTTGATTTTG-3′), and GAPDH probe (FAM-5′-AGCTTCCCGTTCTCAGCCTTCAC-3′-BHQ1). The reaction conditions for RT-dPCR were 40°C for the partition step, 55°C for 10 min (reverse transcription), 95°C for 2 min (initial denaturation), and 45 cycles of 30 s at 95°C (denaturing) followed by 15 s at 58°C (annealing and extension), and then the sapphire tips were scanned and analyzed in a Naica Prism3.

### Statistical analysis.

Statistical analyses were performed using GraphPad Prism 7 and 9 software, and a *P* value of <0.05 was considered significant. Changes in cytokines and chemokines were analyzed by the two-tailed paired *t* test.
